# The Effect of Carbogen Breathing on ^18^F-FDG Uptake in Non-Small-Cell Lung Cancer

**DOI:** 10.1155/2019/2920169

**Published:** 2019-11-22

**Authors:** Yu Lin, Ying-Na Bao, Cong-Xiu Huang, Ji-Hong Zhang, Zhi-Long Yu, Ye Tian, Xiang-Cheng Wang, Yi-Tong Cui

**Affiliations:** ^1^Department of Radiotherapy & Oncology, The Second Affiliated Hospital of Soochow University, Institute of Radiotherapy & Oncology, Soochow University, 1055 Sanxiang Road, Suzhou, Jiangsu 215004, China; ^2^Department of Radiation Oncology, The Affiliated Hospital of Inner Mongolia Medical University, 1 N. Tongdao Street, Hohhot, Inner Mongolia 010059, China; ^3^Department of Nuclear Medicine, Affiliated Hospital of Inner Mongolia Medical University, Hohhot, Inner Mongolia 010050, China; ^4^Department of Medicine, Clinical Medicine, Dushuhu Campus, Soochow University, 199 Ren'ai Road, Suzhou, Jiangsu 215004, China

## Abstract

It has been reported that ^18^F-FDG uptake is higher in hypoxic cancer cells than in well-oxygenated cells. We demonstrated that ^18^F-FDG uptake in lung cancer would be affected by high concentration oxygen breathing. *Methods*. Overnight fasted non-small-cell lung cancer A549 subcutaneous (s.c.) xenografts bearing mice (*n* = 10) underwent ^18^F-FDG micro-PET scans, animals breathed room air on day 1, and same animals breathed carbogen (95% O_2_ + 5% CO_2_) on the subsequent day. In separated studies, autoradiography and immunohistochemical staining visualization of frozen section of A549 s.c. tumors were applied, and to compare between carbogen-breathing mice and those with air breathing, a combination of ^18^F-FDG and hypoxia marker pimonidazole was injected 1 h before animal sacrifice, and ^18^F-FDG accumulation was compared with pimonidazole binding and glucose transporter 1 (GLUT-1) expression. *Results*. PET studies revealed that tumor ^18^F-FDG uptake was significantly decreased in carbogen-breathing mice than those with air breathing (*P* < 0.05). Ex vivo studies confirmed that carbogen breathing significantly decreased hypoxic fraction detected by pimonidazole staining, referring to GLUT-1 expression, and significantly decreased ^18^F-FDG accumulation in tumors. *Conclusions*. High concentration of O_2_ breathing during ^18^F-FDG uptake phase significantly decreases ^18^F-FDG uptake in non-small-cell lung cancer A549 xenografts growing in mice.

## 1. Introduction

Lung cancer is the leading cause of cancer death worldwide [1]. ^18^F-Fluorodeoxyglucose (^18^F-FDG) is an analog of glucose, and ^118^F-FDG positron emission tomography (PET) imaging has emerged as an important clinical tool for cancer detection, staging, and monitoring of response and is routinely used in the clinical management of lung cancer and several other cancer types [2–9].

Warburg effect has been adapted to explain ^18^F-FDG accumulation in cancer cells; increase in glucose metabolism is considered as one of the fundamentals of cancer [10], and a recent observation has demonstrated that Warburg effect hypothesis may not fully explain the difference in ^18^F-FDG uptake between well-oxygenated cancer cells and hypoxic cancer cells logically [11–13]. ^18^F-FDG uptake in terms of glucose metabolism in hypoxic cancer cells has been extensively studied in cell cultures and in animal models of cancers, and it has been reported that hypoxic cancer cells or tissues frequently accumulate higher levels of ^18^F-FDG than well-oxygenated tumor tissues or cells [14–20]. Accordingly, tumor oxygen level may play a key role for ^18^F-FDG uptake in cancer.

Hypoxia reportedly plays a key role in radiotherapy resistance; to overcome hypoxic resistance, multiple attempts had been done to improve tumor tissue oxygen level and, subsequently, improve therapeutic effect [21–24], albeit the literatures were mixed. Investigators have observed the effect of carbogen breathing (95% O_2_ + 5% CO_2_) on tissue oxygen status in cancer, using double hypoxic markers technique. Ljungkvist et al. reported a significant decrease in hypoxia fraction in head-neck cancer models in mice [25], and this finding was reproduced in cancer models from various cancer types by investigators from other laboratories. It is also reported in head-neck cancer and colon cancer models growing in nude mice that carbogen breathing significantly decreased ^18^F-FDG uptake [22–27].

Clinically, it is not surprising for lung cancer patients to become oxygen dependent during the anticancer therapy because of the underlying chronic respiratory disease; lung cancer patients are frequently associated with chronic lung diseases, especially for tobacco smokers. In such patients, the management of therapeutic effect may be affected if ^18^F-FDG uptake is altered because of oxygen breathing.

We hypothesized that high concentration oxygen breathing would affect ^18^F-FDG uptake in lung cancer. Here, we reported the use of correlative imaging methodologies and PET technique to investigate if ^18^F-FDG uptake in lung cancer was altered by carbogen breathing, which potentially affects ^18^F-FDG PET/CT-based cancer management.

## 2. Materials and Methods

We used 7-week-old female nude mice purchased from the Shanghai Institute of Cell Biology to generate lung cancer xenografts. The animal protocols were approved by the Institutional Animal Care and Use Committees of Inner Mongolia Medical University; mice were maintained and used according to the institutional guidelines, and animals were housed five per cage and kept in the institutional small animal facility at a constant temperature and humidity.

In this study, the human non-small-cell lung cancer A549 cells from ATCC were used to generate cancer models. A549 cells were maintained in F-12K medium supplemented with 10% fetal bovine serum, 1% glutamine, and 1% antibiotics. Cells were grown in a humidified incubator at 37°C and 5% carbon dioxide. Cell cultures were harvested with 0.25% (w/v) Trypsin-0.53 mM EDTA solution and washed and suspended in phosphate-buffered saline (PBS).

Subcutaneous tumors were initiated by injecting 5 × 10^6^ tumor cells (suspending in 0.1 ml PBS) subcutaneously into the mouse flank. Experiments were performed when tumors reached approximately 1 cm in diameter.

All animals were imaged in a prone position using Siemens Inveon Micro-CT/PET (Siemens Medical Solutions, Knoxville, TN) system. The PET scanner has a transaxial field of view of 8 cm and an axial field of view of 5.3 cm. The resulting list-mode data were sorted into two-dimensional histograms by Fourier rebinning, and the images were reconstructed by an iterative reconstruction algorithm into a 128 × 128 matrix in 63 slices. Animals were anesthetized by inhalation of an isoflurane (1.5%)-air mixture and maintained throughout PET scan; acquisition time was fixed to 10 min for each PET scan. Siemens Inveon Research Workplace software (IRW Version 3.0) was used for micro-PET image analysis. All image sets for each animal were visually examined using a rotating (cine) three-dimensional display. The window and level settings were adjusted for best intratumoral distribution visibility. Regions of interest (ROIs) were manually drawn, and the mean and maximal activities were recorded from the entire ROIs. The percentage injection dose per gram (%ID/g) was used to represent tissue ^18^F-FDG uptake.

The hypoxia marker pimonidazole hydrochloride (Hypoxyprobe Inc.) was dissolved in physiological saline at a concentration of 20 mg/ml. The blood perfusion marker Hoechst 33342 (Sigma-Aldrich, St. Louis, MO) was dissolved in physiological saline at a concentration of 5 mg/ml.

All animals were fasted overnight before experiments. For PET studies, in case of air breathing, 10 mice were injected via the tail vein with a mixture of ^18^F-FDG (14.8 MBq) and whole body PET scans were performed. To observe the effect of carbogen breathing on ^18^F-FDG uptake, 24 hours after air breathing experiment, the same mouse was placed in a sealable plastic chamber (10 × 10 × 10 cm) into which carbogen was delivered at a flow rate of 5 L/min; one hour after carbogen breathing, animals were injected with ^18^F-FDG (14.8 MBq) and then returned to the carbogen chamber for another hour, and PET scans were followed.

For immunohistochemistry and autoradiography studies, in air breathing, mice were injected via the tail vein with a mixture of ^18^F-FDG (14.8 MBq) and pimonidazole (2 mg) 1 hour before sacrifice, and Hoechst 33342 of 0.5 mg in 0.1 ml was injected via the tail vein 1 min before sacrifice. To observe the effect of carbogen breathing on ^18^F-FDG uptake, mice were placed in a sealable plastic chamber (10 × 10 × 10 cm) into which carbogen was delivered at a flow rate of 5 L/min. After one hour of breathing carbogen, animals were injected with the mixture of ^18^F-FDG/pimonidazole and then returned to the carbogen chamber for another hour prior sacrifice.

Immediately after animal sacrifice, tumor tissue was removed and then frozen and embedded in optimal cutting tissue medium (Sakura Finetek, Torrance, CA), and 5 contiguous 7 *μ*m thick tissue sections were cut using a 3050S cryostat microtome (Leica, Inc).

^18^F-FDG digital autoradiography (DAR) was obtained by placing the tumor sections in a film cassette against an imaging plate. The plate was exposed overnight and read by a Cyclone Plus imaging system (PerkinElmer, Inc). Regions of interest (100 × 100 *μ*m) were drawn over hypoxic cancer cell regions, oxic cancer cell regions, and stroma in digital autoradiography by referring to the immunohistochemical and hematoxylin and eosin stain images obtained from same section. Digital autoradiography imaging was quantified by an OptiQuant software (PerkinElmer Inc.), and ^18^F-FDG uptake in each microenvironment component was expressed as percentage injected dose per gram tumor tissue (%ID/g) [20].

Images of the distributions of pimonidazole, GLUT-1, and Hoechst 33342 were obtained in tumor sections after the completion of ^18^F-FDG digital autoradiography exposures as described [27]. In order to minimize issues associated with section alignment and registration, the same tumor section used for DAR or contiguous adjacent sections were used for all images. Briefly, slides were air-dried, fixed in cold acetone (4°C) for 20 min, and incubated with SuperBlock (Pierce Biotechnology, Rockford, IL) at room temperature for 30 min. All antibodies were also applied in SuperBlock. Sections were then incubated with FITC-conjugated anti-pimonidazole monoclonal antibody (Hypoxyprobe Inc), diluted 1 : 25, for one hour at room temperature. GLUT-1 staining was performed either on the same section or on the adjacent section to that stained for pimonidazole by incubating for one hour at room temperature with rabbit anti-GLUT-1 polyclonal antibody (Millipore) diluted 1 : 50. Sections were washed three times in PBS and incubated for one hour at room temperature with AlexaFluor568-conjugated goat anti-rabbit antibody (1 : 100, Molecular Probes, Eugene, OR). Images were acquired at 100× magnification using an Olympus BX40 fluorescence microscope (Olympus America Inc., Melville, NY) equipped with a motorized stage (Prior Scientific Instruments Ltd., Cambridge, UK). Hoechst 33342 and pimonidazole were imaged using blue and green filters, respectively. GLUT-1 was imaged using a red filter [27].

After the acquisition of fluorescence images, tumor sections were stained with hematoxylin and eosin (H&E) and imaged by light microscopy. Microscopic images were coregistered and analyzed using Adobe Photoshop 7.0 (Adobe, San Jose, CA).

Statistical significance was examined by two-tailed Student's *t*-test. A *P* value less than 0.05 was considered as statistically significantly different.

## 3. Results

**Figure 1:**
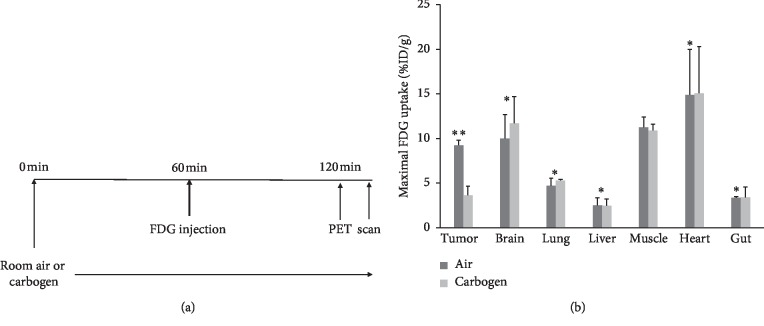
(a). PET study protocol. (b) Comparing ^18^F-FDG uptake in A549 subcutaneous xenografts under carbogen (95% O_2_ + 5% CO_2_) breathing condition versus mice breathing room air. Carbogen breathing significantly decreases ^18^F-FDG uptake. ^*∗∗*^*P* < 0.05; ^*∗*^not statistically significant; *n* = 10.

PET studies were performed on subsequent day basis (Figure 1(a)). ^18^F-FDG uptake in A549 subcutaneous tumor in mice breathing carbogen was significantly lower than that in mice breathing air, and carbogen breathing little affected ^18^F-FDG uptake in normal organs (Figure 1(b)).

**Figure 2:**
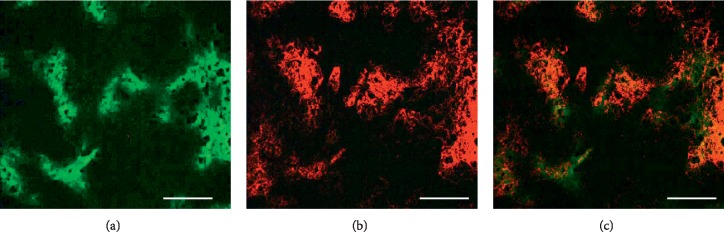
Immunohistochemical visualization of hypoxia in A549 subcutaneous xenografts by Pimonidazole and GLUT-1 staining under room air breathing condition. (a) Hypoxia detected by pimonidazole binding (green). (b) Hypoxia detected by GLUT-1 (red). (c) Overlay imaging shows that pimonidazole binding (green) and GLUT-1 expression (red) are generally colocalized. Both scale bars = 0.2 mm; a total of 10 tumors were examined.

**Figure 3:**
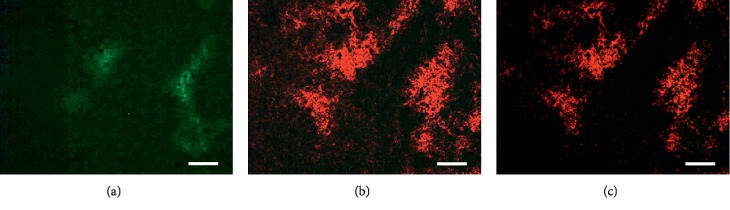
Immunohistochemical visualization of hypoxia in A549 subcutaneous xenograft by pimonidazole and GLUT-1 staining under carbogen breathing condition. (a) Hypoxia detected by pimonidazole binding (green). (b) Hypoxia detected by GLUT-1 (red). (c) Overlay imaging shows little pimonidazole binding (green) with GLUT-1 overexpression. Scale bars = 0.2 mm. A total of 6 tumors were examined.

In immuohistochemical study, when mice breathed air, there was spatial colocalization between high levels of pimonidazole binding and GLUT-1 expression in A549 subcutaneous xenografts (*n* = 10), which indicated that, under air breathing, all A549 subcutaneous xenografts we examined contained regions of hypoxia; representative images are shown in Figure 2. In contrast, when animals breathed carbogen, there was a major reduction in pimonidazole binding in regions of tumor with higher GLUT-1 expression in similar size of A549 subcutaneous xenografts (*n* = 6), indicating that previous hypoxia had been oxygenated because of oxygen breathing treatment; representative images are presented in Figure 3.

**Figure 4:**
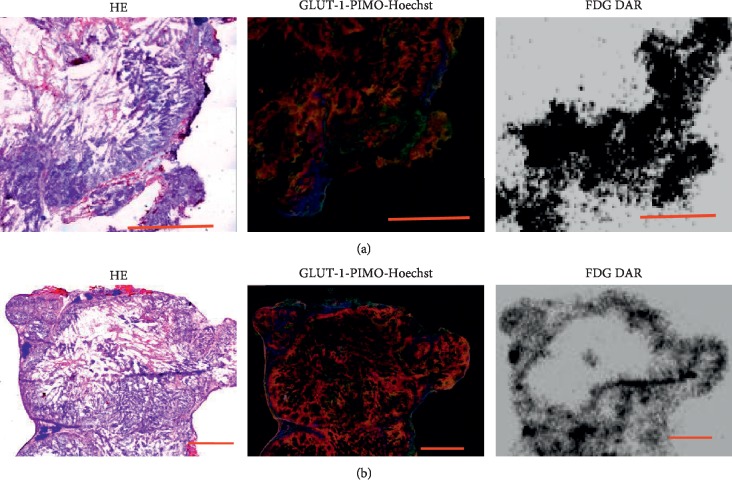
Effect of carbogen breathing on ^18^F-FDG uptake in regions with GLUT-1 overexpression. (a) Under air breathing condition, higher ^18^F-FDG accumulation, pimonidazole binding, and GLUT-1 are colocalized. (b) Under carbogen breathing, decrease in ^18^F-FDG accumulation and lower pimonidazole binding are found in GLUT-1 overexpressing regions, and Hoechest 33342 binding is not affected indicating no blood perfusion change. H&E: hematoxylin and eosin; GLUT-1-PIMO-Hoechst: overlay of GLUT-1 (red), pimonidazole (green), and Hoechst 33342 (blue); DAR: ^18^F-FDG digital autoradiography; scale bars = 2 mm.

In digital autoradiography, when 5 mice were treated with carbogen breathing, glucose demand in A549 tumors as measured by ^18^F-FDG uptake was 4.19 ± 1.32%, which was significantly lower than that in mice breathing air (9.67 ± 4.35, *n* = 5 mice, *P* < 0.01), and pimonidazole binding decreased in high GLUT-1 expression regions when animals breathed carbogen (Figure 4).

## 4. Discussion

^18^F-FDG was initially used for imaging two volunteers in 1970s. ^18^F-FDG PET has been used for cancer management following a variety of therapies, in addition to its use for cancer and stage detection [2–9]. Clinically, ^18^F-FDG plays a key role in monitoring anticancer therapy effect and lung cancer detection, though false positivity in tuberculosis and other chronic infections and false negativity in some slowly growing adenocarcinomas weaken the weight of cancer detection with^18^F-FDG PET. However, it has been well established that ^18^F-FDG PET has successfully observed therapeutic effect following treatments; that is, if in baseline PET study, ^18^F-FDG accumulated malignancy decrease in ^18^F-FDG uptake in a following ^18^F-FDG PET after treatment is the sign of malignancy response to the therapy. Factors that affect ^18^F-FDG uptake may interrupt the ^18^F-FDG PET assessment of therapeutic effect. In this study, carbogen breathing significantly decreased ^18^F-FDG uptake in lung cancer (Figures 1 and 4); therefore, high concentration of oxygen breathing does affect the therapeutic management.

Carbogen breathing or high concentration oxygen is routinely prescribed to patients who develop respiratory dysfunction because of chronic lung diseases. It is not uncommon for lung patients to become oxygen dependent [28, 29]. If possible, high concentration breathing should be avoided right prior ^18^F-FDG PET study.

The effect of carbogen breathing in altered tumor hypoxia status has been observed in lung cancer model (Figures 2–4), in colon cancers, head-neck cancer, and other cancer types [22–27].

Changes in tumor hypoxia can be detected using double hypoxic markers [25, 30]. The principle of this method is that one marker represents previous hypoxia and the other one represents the current hypoxia. With exogenous hypoxic markers such as the 2-nitroimidazole compounds pimonidazole, the two markers EF5 and CCI-103F are administered separately. Assays based on endogenous hypoxia-regulated protein such as HIF1*α*, carbonic anhydrase 9(CA9), and GLUT1 can also be used [30]. These rely on differences in the protein lifetime so that current hypoxia (e.g., HIF1*α* distribution) can be compared with “historic” hypoxia (e.g., CA9 distribution). In addition, exogenous and endogenous tracers can be combined in an appropriate manner so that current hypoxia and previous hypoxia can be visualized [30].

In this study, we used GLUT-1 and pimonidazole dual-hypoxia markers technique: GLUT-1 reflected the historic hypoxia, and pimonidazole binding represents the hypoxic status following carbogen breathing treatment. Following two hours of carbogen breathing (one hour before and one hour after ^18^F-FDG administration), there was a major reduction in both ^18^F-FDG uptake and pimonidazole binding in hypoxic regions (which was stained positive for GLUT-1) compared to the mice breathing room air (Figures 2–4). Carbogen-breathing mediated-oxygenation produced rapid decrease in tumor ^18^F-FDG uptake raises concerns with a potential clinical significance. In particular, interpretation difficulties may arise when the changes in ^18^F-FDG uptake are being used to monitor the tumor's response to therapy since the hypoxic status changes in the tumor induced by the therapy may be challenged when the patient becomes oxygen dependent [31].

We and others have observed that carbogen breathing treatment significantly decreased ^18^F-FDG uptake in subcutanous xenografts tumors of a variety of cancer types [22–27] (Figures 1 and 4), and human study urges to be done confirm that what we found in animals also applies to patients with lung cancer. Before human studies investigating high concentration effect on ^18^F-FDG uptake are concluded, we suggest the suspension of high oxygen breathing for patients for couple hours before ^18^F-FDG PET study if possible.

Although Warburg effect theory is widely accepted as an explanation for ^18^F-FDG PET oncology application, our data indicate that change in oxygen status of tumor tissue may change glucose utility in cancer cells which cannot be explained by Warburg effect but would be logically explained by Pasteur effect theory.

In conclusion, high concentration of O_2_ breathing during ^18^F-FDG uptake phase may significantly blunt ^18^F-FDG uptake; thus, in cancer patients, this may decrease the accuracy of the FDG PET/CT for the detection, staging of the disease, and the assessment of the therapy effect following anticancer therapy.

## Data Availability

The data used to support the findings of this study are available from the corresponding author upon request.
